# Sample size optimisation when designing sampling plan for risk assessment relating to adventitious presence of allergens in food

**DOI:** 10.1186/2045-7022-3-S3-P137

**Published:** 2013-07-25

**Authors:** CF Elegbede, A Papadopoulos, A Crépet

**Affiliations:** 1 UMERPC, ANSES, Maisons-Alfort, France

## Background

Despite the avoidance of allergen-containing products by allergic individuals, a risk of allergic reaction to food allergen still remains. These contaminations may result from the shared use of processing equipment in the food industry, and are not always identified. It is therefore important to select a representative food sample to analyse food contamination and to further assess risk for persons who have an allergy to specific food. The optimal sample size determination is one of the most recurrent problems when designing a sampling plan in every experiment. Difficulties are especially observed when the studied population may be divided into several groups. That is the case when analysing adventitious contamination of food product consumed by allergic population. Indeed, over 150 different foods have been reported to cause food-allergic reactions (Hefle, Nordlee et al. 1996), and some foods are found more involved in allergic reactions than others. This work was a part of the project MIRABEL which aims to set an integrated and operational framework for the allergic risk analysis, in order to improve quality of life of allergic sufferers.

## Methods

This work was performed within three steps. Firstly, some foods products which are a fair reflection of the allergic population food consumption behaviour were chosen. Indeed, since specific avoidance diet recommended by allergists, allergic suffers was supposed to have specific consumption behaviours towards foods which could contain allergen. Thus, in the second step, various criteria based on: (i) allergic population food consumption behaviour, (ii) knowledge on contamination of each food product group, and (iii) their allergen concentration levels, were defined. Finally, a statistical model combining these criteria was proposed to assess adequate sample size.

## Results

The defined model was used to characterise food contamination to analyse the risk of peanut allergy. Peanut is one of the most common allergen, which can be adventitiously present in various foodstuffs. In this view, food consumption behaviour of peanut allergic population obtained from MIRABEL program was considered, and optimal sample size was estimated for each group of defined food product.

## Conclusion

We defined a tool to improve food allergy management and further assess risk relating to adventitious presence of allergens in foods.

## Disclosure of interest

None declared.
